# Usefulness of the ripple map and late annotation mapping to visualize an activation pattern within Koch's triangle in a patient with persistent left superior vena cava

**DOI:** 10.1002/joa3.70030

**Published:** 2025-03-04

**Authors:** Takashi Kanda, Hitoshi Minamiguchi, Riku Iwami, Osamu Iida, Yoshiharu Higuchi

**Affiliations:** ^1^ Cardiovascular Division, Advanced Cardiac Rhythm Management Center Osaka Keisatsu Hospital Osaka Japan; ^2^ Cardiovascular Division Osaka Keisatsu Hospital Osaka Japan

**Keywords:** atrioventricular nodal reentrant tachycardia, high‐density mapping, Koch's triangle, persistent left superior vena cava, slow pathway

## Abstract

High‐resolution mapping with Ripple and LAM modules enabled precise identification of slow pathway ablation targets in a PLSVC patient. This novel approach overcame anatomical challenges, offering a more effective strategy for AVNRT treatment in complex cases.

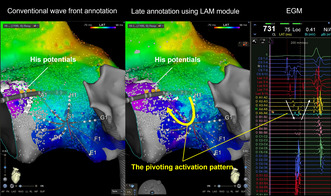

Persistent left superior vena cava (PLSVC) is a rare vascular anomaly that can complicate slow pathway (SP) ablation procedures because of the altered anatomy of Koch's triangle. Recent advancements in SP ablation have highlighted the importance of detailed conduction mapping within Koch's triangle for effective treatment.^1^, ^2^, ^3^, ^4^ This report presents a novel approach utilizing high‐resolution, high‐density mapping with ripple mapping and late annotation mapping (LAM) modules to visualize activation patterns within Koch's triangle in a patient with PLSVC. This method allowed precise identification of the ablation target, overcoming the challenges posed by the complex anatomy.

Here is an example of the clinical course of a representative case. A 75‐year‐old male, with symptomatic persistent atrial fibrillation, was admitted for radiofrequency (RF) ablation. An echocardiogram and chest CT confirmed the presence of PLSVC. Catheter ablation of the persistent atrial fibrillation was performed using the CARTO 3 (Biosense Webster, Irvine, CA, USA) system, QDOT MICRO (Biosense Webster) catheter for ablation, and Octaray 3‐3‐3 (Biosense Webster) catheter for mapping of the pulmonary veins and PLSVC. After bilateral pulmonary vein isolation and PLSVC isolation, intracardiac cardioversion was performed with 10 J, and sinus rhythm was restored. A subsequent electrophysiological examination demonstrated the occurrence of a narrow QRS tachycardia. The tachycardia started with a jump up, and a diagnosis of typical AVNRT was made based on the electrophysiological findings. The PLSVC was contrasted to obtain the anatomical information, but it was difficult to accurately identify Koch's triangle because of the huge PLSVC (Figure 1).

**FIGURE 1:**
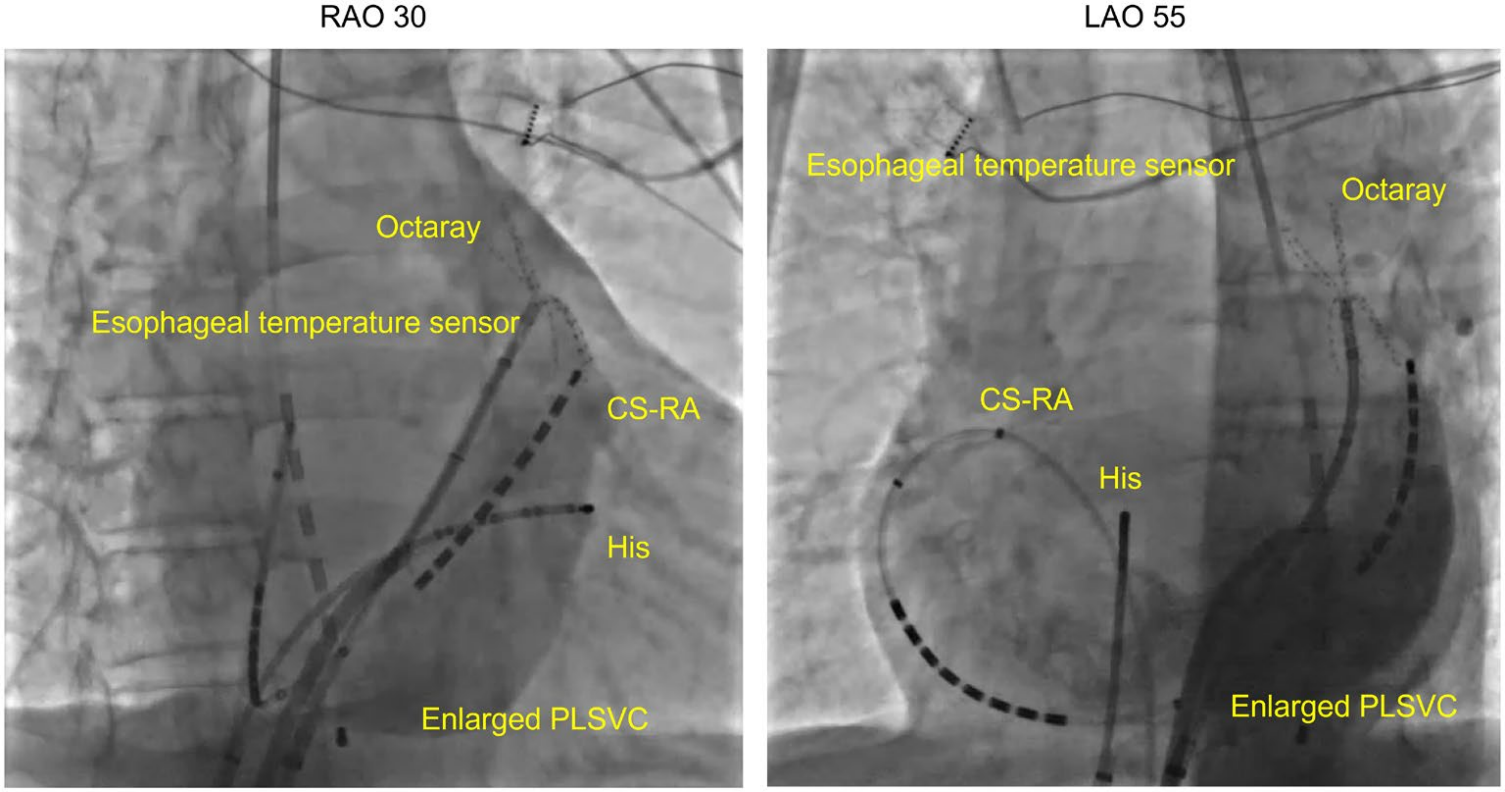
Venography of the PLSVC. Contrast medium was injected through a sheath inserted in the PLSVC. The PLSVC was extremely enlarged, making it difficult to accurately assess the anatomy around Koch's triangle. PLSVC, persistent left superior vena cava.

Therefore, we proceeded with high‐density mapping of the right atrial septum using the Octaray catheter during sinus rhythm to create a local activation map of the atrial septum. When displayed with conventional wavefront annotation, Koch's triangle was only excited from upper to lower. However, with the ripple map and late annotation mapping, a discrete late atrial activation zone was identified along the tricuspid annulus, which was associated with a fractionated corresponding atrial electrogram (Figure 2, Videos S1 and S2). Those conduction patterns with a pivoting excitation within Koch's triangle represented the slow pathway ablation target. A single radiofrequency application to that area led to the occurrence of accelerated junctional beats. Several additional applications were delivered from the first application point to the tricuspid annulus to obtain non‐inducibility and to successfully eliminate the SP. The patient was discharged on the third day. AF and AVNRT did not recur during the 6 months following ablation.

**FIGURE 2:**
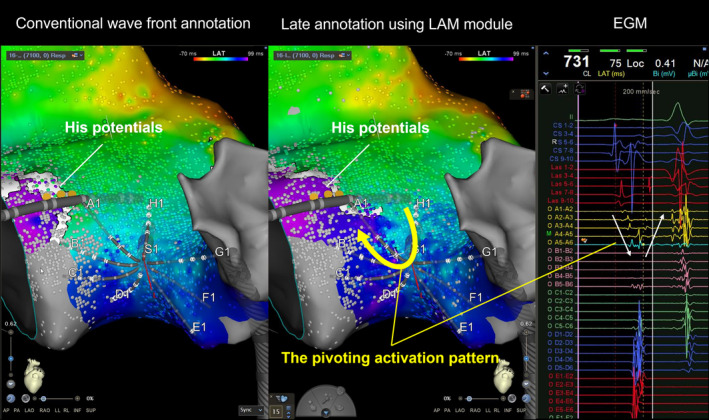
High‐density and high‐resolution mapping within Koch's triangle. In the LAT map created by the conventional wavefront annotation, the flow appears from above to below near the tricuspid valve. However, in the LAT map with a late annotation, the functional line of block and the pivoting point were identified, and the excitation was observed to ascend the tricuspid valve annulus up to the His potential recording site. LAT, local activation time.

Although several methods have been reported in recent years to evaluate the conduction within Koch's triangle and delineate typical AVNRT treatment targets, to our knowledge, this is the first report to examine the conduction within Koch's triangle in detail using a ripple map and LAM module. In our opinion, this approach is considered more effective in patients with a complex anatomy complicated by PLSVC who may be difficult to treat with conventional techniques.

This case demonstrates the successful treatment of AVNRT in a patient with PLSVC, where anatomical challenges made conventional ablation approaches difficult. High‐density, high‐resolution mapping within Koch's triangle, combined with Ripple mapping and LAM, enabled precise identification of the ablation target.

Recent studies have emphasized the importance of detailed conduction mapping for SP ablation. Kumagai et al.^2^ utilized HD Grid mapping catheters (Abbott) and Ensite X (Abbott) to identify pivoting activation patterns around block lines within Koch's triangle in patients with dual pathway physiology. Wakamatsu et al.^3^ used Rhythmia HDx (Boston Scientific) and Orion catheters to highlight fragmented potentials during tachycardia with the LUMIPOINT module. Gerontitis et al.^5^ used a Pentaray (Biosense) or Orion in PLSVC cases and found that SP ablation targets were consistently identified by an area in the right atrial septum with the latest activation time and multicomponent atrial electrograms adjacent to a region with isochronal crowding.

A high‐density, high‐resolution map is necessary to accurately determine the target area. In this case, more than 7000 points were acquired and analyzed only in Koch's triangle, and the ripple map and LAM module enabled an accurate determination of the target site without manual annotation. In conventional wavefront annotation, the annotation was placed on the first component of the double potential. However, the LAM module annotated the second component, allowing for a clearer recognition of conduction downstream of the pivoting point. Additionally, since the Ripple map can represent excitation independent of annotation, delayed potentials could be visualized, as demonstrated in Video S2. It is impossible to analyze a huge number of potentials one by one, and the fact that manual annotation is not required is clinically quite important.

In addition, in this case, we performed ablation of AVNRT in addition to atrial fibrillation ablation with mapping in Koch's triangle using an Octaray 3‐3‐3. We considered this choice to have been successful as a result. When using paddle‐type catheters, it is often difficult to fit the catheter on Koch's triangle and the surrounding uneven surfaces. Also, basket‐type catheters have electrodes on a sphere, so all electrodes cannot be in contact with the myocardium, limiting the area that can be mapped at one time. The Octaray catheter, on the other hand, allows each of the splines to be softly placed. In addition, the Octaray catheter has longer splines and more electrodes than the Pentaray catheter, allowing mapping of a wider area and recording the electrograms around the pivoting point all at one time, as shown in Figure 2.

Finally, since the conventional approach was difficult in a PLSVC case, mapping within Koch's triangle was important, and the annotation‐independent ripple map and LAM module, which could evaluate delayed potentials, were useful for visualizing the electrograms associated with the SP.

High‐resolution, high‐density mapping using a ripple map and LAM module around Koch's triangle significantly enhanced the precision in accurately determining the target site for SP ablation in patients with PLSVC.

## FUNDING INFORMATION

None.

## CONFLICT OF INTEREST STATEMENT

The other authors have no conflicts of interest to disclose.

## ETHICS STATEMENT

The study was approved by the Institutional Review Board of our institution, and written informed consent was obtained from all individual participants included in the study.

## Supporting information


Video S1.

Video S2.

## References

[1] Bailin SJ , Rhodes TE , Arter JC , Kocherla C , Kaushik N . Physiology of slow pathway conduction during sinus rhythm: evidence from high density mapping within the triangle of Koch. J Interv Card Electrophysiol. 2022;63:573–580. 34518928 10.1007/s10840-021-01061-4

[2] Kumagai K , Toyama H . Activation pattern within Koch's triangle during sinus rhythm in patients with and without atrioventricular nodal reentrant tachycardia. J Interv Card Electrophysiol. 2024;67(1):139–146. 10.1007/s10840-023-01589-7 37311982

[3] Wakamatsu Y , Nagashima K , Kaneko Y , Mori H , Tsutsui K , Maegaki M , et al. Novel ablation strategy targeting the slow pathway visualized by ultrahigh‐resolution mapping in typical slow‐fast atrioventricular nodal reentrant tachycardia. Circ Arrhythm Electrophysiol. 2023;16:e011497. 36799216 10.1161/CIRCEP.122.011497

[4] Watanabe T , Hachiya H , Watanabe H , Anno K , Okuyama T , Harunari T , et al. Relationship between the atrial‐activation pattern around the triangle of Koch and successful ablation sites in slow‐fast atrioventricular nodal reentrant tachycardia. J Arrhythm. 2024;40:363–373. 38586857 10.1002/joa3.12999 PMC10995602

[5] Gerontitis D , Pope MTB , Elmowafy M , Sadagopan S , Yue AM . High‐density electroanatomic activation mapping to guide slow pathway modification in patients with persistent left superior vena cava. Heart Rhythm. 2023;20:1018–1025. 37019166 10.1016/j.hrthm.2023.03.1537

